# Using Automated Speech Processing for Repeated Measurements in a Clinical Setting of the Behavioral Variability in the Stroop Task

**DOI:** 10.3390/brainsci13030442

**Published:** 2023-03-04

**Authors:** Terje B. Holmlund, Alex S. Cohen, Jian Cheng, Peter W. Foltz, Jared Bernstein, Elizabeth Rosenfeld, Bruno Laeng, Brita Elvevåg

**Affiliations:** 1 Department of Clinical Medicine, University of Tromsø—The Arctic University of Norway, 9037 Tromsø, Norway; 2 Department of Psychology, Louisiana State University, Baton Rouge, LA 70803, USA; 3 Analytic Measures Inc., Palo Alto, CA 94301, USA; 4 Institute of Cognitive Science, University of Colorado Boulder, Boulder, CO 80309, USA; 5 Department of Psychology, University of Oslo, 0315 Oslo, Norway; 6 Norwegian Centre for eHealth Research, University Hospital of North Norway, 9038 Tromsø, Norway

**Keywords:** Stroop, automatic speech recognition, mobile, cognitive control, psychiatry

## Abstract

The Stroop interference task is indispensable to current neuropsychological practice. Despite this, it is limited in its potential for repeated administration, its sensitivity and its demands on professionals and their clients. We evaluated a digital Stroop deployed using a smart device. Spoken responses were timed using automated speech recognition. Participants included adult nonpatients (N = 113; k = 5 sessions over 5 days) and patients with psychiatric diagnoses (N = 85; k = 3–4 sessions per week over 4 weeks). Traditional interference (difference in response time between color incongruent words vs. color neutral words; M = 0.121 s) and facilitation (neutral vs. color congruent words; M = 0.085 s) effects were robust and temporally stable over testing sessions (ICCs 0.50–0.86). The performance showed little relation to clinical symptoms for a two-week window for either nonpatients or patients but was related to self-reported concentration at the time of testing for both groups. Performance was also related to treatment outcomes in patients. The duration of response word utterances was longer in patients than in nonpatients. Measures of intra-individual variability showed promise for understanding clinical state and treatment outcome but were less temporally stable than measures based solely on average response time latency. This framework of remote assessment using speech processing technology enables the fine-grained longitudinal charting of cognition and verbal behavior. However, at present, there is a problematic lower limit to the absolute size of the effects that can be examined when using voice in such a brief ‘out-of-the-laboratory condition’ given the temporal resolution of the speech-to-text detection system (in this case, 10 ms). This resolution will limit the parsing of meaningful effect sizes.

## 1. Introduction

The Stroop color-word interference task [1,2] is commonly regarded as one of the “gold standard” cognitive measures of attentional control and its biases [3]. Although different versions exist in the literature, with variations in the number of stimuli, sensory domain of stimulus, response format, and summary performance measures, the canonical version consists of naming the ink color of a printed word while ignoring the meaning of the word itself. If the printed word and its color are incongruent (e.g., the word **RED** printed in blue ink), the over-learned automated process of reading the word interferes and produces a hypothesized conflict cost, resulting in increased errors and in fewer trials being completed. Digital versions of the Stroop have been used in research for decades and allow for more sensitive evaluation of response time and interference effects in milliseconds. By careful adjustment of stimulus material, the approach has also been used for mapping attentional biases in, for example, depression, anxiety, post-traumatic stress disorder and phobias [4,5] as well as for substance use disorders [6] and schizophrenia [7,8]. Recent technical innovations allow for mobile and automated digital Stroop versions. The present project establishes proof-of-concept for this approach using automated speech recognition for computing response time speed and accuracy.

The use of a mobile digital Stroop has at least four major advantages over traditional versions. First, it offers a more inexpensive administration. Cognitive disorders reflect one of the costliest maladies in the developed world, and traditional cognitive assessment is time intensive for both patients and expert administrators [9]. Thus, a mobile automated version can help reduce expenses and improve cognitive assessment in populations that lack resources or are structurally disadvantaged to access traditional cognitive clinics. Second, a digital platform for Stroop testing can offer flexibility that allows for a range of ways to probe cognitive processes and biases. However, it remains to be established whether a mobile and automated assay has the sensitivity to reliably capture the traditional facilitation and interference effects. Distinguishing between these effects has been important in differentiating between generalized slowing and putative deficits in cognitive control. Ultimately, this can create a tool that can probe different aspects of behavioral performance to understand mechanistically and functionally distinct disease entities (sufficient resolution, see [10,11]). Third, a digital Stroop facilitates novel metrics beyond simply accuracy used in traditional administrations. Being able to measure trial-level interference effects rather than inferring these effects from global performance offers greater sensitivity. The use of trial-level timestamping of the start and end of speech responses can further facilitate a variety of metrics, among these the duration of the spoken utterance, an established parameter for word stress level [12]. Another metric it enables is Intra-Individual Variability of responses, a measure with few assumptions about the origin of performance effects (e.g., specific word-type effects) but has proven useful for the assessment of overall cognitive function and comes with plausible neurobiological underpinnings [13]. Finally, a mobile automated digital Stroop test could facilitate longitudinal and remote tracking over time. Tracking changes in cognitive abilities over time can provide critical information for evaluating neurodegenerative and psychiatric disorders and for evaluating treatment response. For example, measuring over periods of months and years could help in the differential diagnosis between neurodegeneration and healthy aging and could help optimize pharmacological and rehabilitative treatments designed to mitigate cognitive dysfunction. Measuring over days and weeks could help optimize sleep schedule, reduce anxiety, evaluate psychosis, and otherwise optimize cognitive functioning for a variety of concerns in neurotypical, psychiatric and neurological populations.

To date, there have been other successful implementations of the Stroop test using mobile devices for data collection [14,15,16], but these implementations involved responding by pressing on-screen buttons (e.g., buttons labeled “red”, “blue”, etc.). Using buttons complicates the response process by demanding a visual scan of the screen to locate the correct place to press, and in addition, there are technical challenges of making response time measurements related to different sampling rates of different device screens (usually 60 Hz, i.e., ±16.7 ms temporal resolution, may vary between devices). Speech is the original medium of this task and arguably provides the most direct assay of trial-level response performance and of the underlying cognitive processes. Speech responses have typically required manual coding, a potentially laborious effort that now can be done using automated speech recognition.

It remains to be established if the timestamping of spoken utterances using automated speech recognition has sufficient sensitivity to detect the Stroop effect. This sensitivity will depend on temporal accuracy, precision and resolution, where the precision of timestamps will be limited by the resolution for the most fine-grained comparisons of response time performance. Automatic speech recognition typically generates outputs with a temporal resolution of ±10 ms (e.g., a word starts at 1.35 s after the presentation of a stimulus and ends at 1.82 s). This stems from a “moving window” procedure, where a window (typically 25 ms long) is examined for the presence of the speech sounds of a word, then the window is moved 10 ms along for another check, and thus it continues until the recording is over. Can this temporal resolution be sufficient to detect cognitive and clinical effects of interest? Ward [17] provides a useful ‘rule of thumb’, namely that measurement precision should be at least an order of magnitude the size of the difference that is to be detected. The precision, or variable error, of the timestamps of word onsets in a Stroop task is unknown and may depend on procedures/algorithms and variable recording conditions. However, as with the previously mentioned issue of the screen refresh rate, errors of just a few milliseconds will be hidden in the 10 ms “chunks” of timestamp information, so even in a best-case scenario, there will be a 10 ms uncertainty or “quantization error” of when someone responded. Since the size of the classic Stroop color-word interference has been shown to be in the 150 ms range (average 153 ms in three different experiments our methods are based on [18,19,20]), it should therefore be detectable using speech recognition tools. Even for other, more clinically relevant effects, this may be sufficient. For example, words such as “spider” and “crawl” demonstrated 190 ms interference delays in individuals affected by a phobia [5]. While demonstrating the traditional and robust effects can be interesting, the true value of Stroop testing lies in detecting pathological changes within and between individuals. Across the three studies mentioned earlier [18,19,20], the average difference in interference between groups (serious mental illness vs. healthy controls) was a mere 27 ms, and for facilitation, 71 ms. It is, therefore, important to establish whether the common 10 ms temporal resolution of speech-to-text systems and the corresponding limit to the precision of the timestamping can be sufficient for detecting clinically relevant effects.

**Figure 1:**
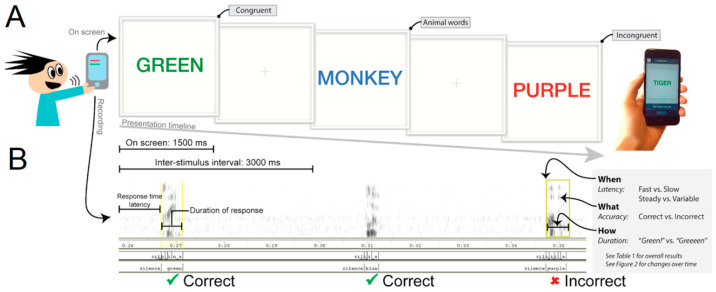
Details on how behavioral data on word category effects can be collected using smart devices. Panel (**A**): Three different stimulus conditions were presented visually in random order on the screen of a smart device, with a total of 32 presentations per testing session. In the example, first is presented a trial with the label “congruent”, where the word **GREEN** is printed in green color. The word remained on the screen for 1.5 s, followed by 1.5 s with a fixation cross, before the presentation of an “animal word” trial with the word **MONKEY** presented in blue color. Last is illustrated an “incongruent” trial, where the word **PURPLE** was presented in red, representing a conflict between ink color and the meaning of the word. Panel (**B**): Spoken responses to naming ink colors were recorded, and automatic speech recognition software detected response latency, duration and accuracy. The file with recorded audio was segmented into either “silence” or the phonemes of the respective responses, making it possible to ignore phonations of hesitations such as “uh”. The timestamp of the signal to flash the stimulus word on the screen was subtracted from the word onset timestamp to measure the response time latency (the “When”). Responses were classified as either “Correct” or “Incorrect” (the “What”), and incorrect responses were not included in the response time analysis. The “How” was indexed by the duration of spoken utterance (e.g., ‘greeeen’ versus ‘green’), a prosodic feature of stress or emphasis.

The present project evaluated a mobile, digital and fully automated Stroop test in a young adult university student sample and in an older sample recruited from an inpatient substance use treatment facility. The latter sample offers potential insight into the unfolding of putative attentional processes as a function of symptom amelioration or exacerbation in treatment. This Stroop test is similar in form and structure to the standardly employed single-trial Stroop with color-congruent, -incongruent and -neutral stimuli (based on the methodology in [18,19,20]), but specifically designed for daily and remote administration (see Figure 1). Sessions with the mobile device lasted around 15 min (1.5 min for the Stroop task) and contained different tasks as part of a larger study on the assessment of language, memory and psychomotor skills, as well as self-report on mental states (see [21] for an overview of the tasks). We evaluated this mobile test in its ability to tap both traditional response time measures, consequent facilitation and interference effects, as well as novel response utterance duration and intra-individual variability measures. We focused on (a) data usability and compliance, (b) test-retest stability over daily assessments, (c) convergence with episodic clinical symptoms (covering a 2-week epoch), (d) convergence with the mental state (assessed at the time of Stroop testing), and (e) convergence with treatment outcome in the patient sample. Given the temporal resolution of 10 ms, it was expected that the traditional Stroop effects should be readily detected, while smaller effects (e.g., in exploratory analyses of differences between groups and clinical states) would be harder to parse.

## 2. Materials and Methods

### 2.1. Participants

We examined the performance of 113 university students (19.8% male, mean age = 20.0, SD = 1.9) as well as the performance of 85 male inpatients (Mean age = 39.1, SD = 11.2) undergoing treatment for substance abuse disorders. Patients had a primary diagnosis of substance abuse, most prevalently with addiction to alcohol (26%), cocaine (26%), and opioids (25%). Additionally, 63% had psychiatric comorbidity, most commonly depression (39%). In light of the notable differences in health and age between healthy and patient participants, we assumed that there would be differences in performance between groups, and therefore a valid measure of attentional control should reveal such a difference (i.e., part of the proof of concept).

The study was approved by the Louisiana State University Institutional Review Board (#3618), and all methods were performed in accordance with the relevant regulations and guidelines. To be included, participants had to (a) be able to legally offer informed consent (e.g., not need and thus not have a legal guardian), (b) choose to offer written informed consent, (c) watch a 3-min instructional video highlighting the risks, rewards and expectations of study participation, and (d) demonstrate understanding of the study verified by passing a quiz with questions about the nature of the study. This informed consent was obtained from all participants. Students were rewarded with course credits for participation, while patients were given monetary rewards of $5 per completed session.

### 2.2. Procedure

Participants were asked to give verbal and touchscreen responses presented on a smart device using an in-house developed mobile application for the iOS operating system from Apple Inc. Each session with the smart device contained one sequence of Stroop task trials. A visual prompt appeared before the sequence commenced, with the words: “SAY TEXT COLOR” and a vocal prompt instructing, “Say the color the word is printed in”. The first word presentation was initiated by the press of a touchscreen button from the user, then all subsequent presentations for the session appeared consecutively in a randomized sequence for 96 s. The paradigm was based on a well-established procedure introduced by Carter, Robertson and Nordahl [18] and used in numerous other studies (e.g., [19,20,22]). For the mobile implementation, we made some notable adjustments. First, the number of trials was reduced to strike a balance between what would be an acceptable duration for an ambulatory task for chronically ill patients and what could produce a sufficiently high number of responses for statistical analysis. The usability aspects of the test development were of critical importance to achieving compliance from participants, as we received feedback during preceding experiments in the study from participants indicating that the duration of testing may have been too long. Second, we increased the pace of the task due to feedback preceding the study proper from users that the task was “sluggish”.

Thirty-two words were presented in three stimulus conditions (eight congruent stimuli, eight incongruent stimuli and 16 animal-word stimuli). Congruent stimuli consisted of color words printed in the same color that they represent, e.g., “**RED**” printed in the red color. Incongruent stimuli consisted of color words printed in one of the remaining three colors (e.g., **RED** printed in green color). For measurement of performance unrelated to color-word congruence, animal words of three to six letters (**DOG, BEAR, TIGER, MONKEY**) were presented in all four colors. Words were presented on a white background in capital letters (Arial bold font, height = 165 pixels) using four different colors: **RED, BLUE, GREEN** and **PURPLE**. Words remained on the screen for 1500 ms, followed by a fixation cross for 1500 ms, resulting in a regular Inter-Stimulus Interval (ISI) of 3000 ms (Figure 1A). All responses recorded within the ISI were defined as a response to the preceding word, and responses after the ISI were thus defined as responses to the next trial.

### 2.3. Clinical and Functioning Measures

Patients were diagnosed as part of the regular clinical procedures at the recruitment site. Clinical ratings were performed using information obtained from medical records, the patients’ treatment teams and self-report and behavioral observations made during the research interview. Factor subscale scores reflecting positive depression/anxiety and mania/excitement symptoms were computed [23]. Preliminary diagnoses and ratings were made by one of four doctoral-level students who were trained to criterion (Intra-class Correlation Coefficient values N 0.70). Final ratings were determined based on consensus from the group of students and their supervisor. The Brief Psychiatric rating scale (BPRS) [24] was used on 49 of the 85 patients to map self-reported psychiatric symptoms in the two-week period before testing commenced. Similarly, the Brief Symptom Inventory (BSI) [25] was used to map psychiatric symptoms for the same two-week period in nonpatients. Furthermore, the mobile application where the Stroop testing was conducted included items where participants self-reported on mental states. This self-report was conducted by moving a sliding marker on the screen between options of agreement or disagreement, giving a score between 0 and 100. We modeled the results for two relevant questions, namely, “Can you concentrate today? (Cannot concentrate = value 0 vs. Steady concentration = value 100), and “Do you feel helpless? (Not helpless = value 0 vs. Very helpless = value 100). As a measure of treatment outcome, whether or not patients completed the treatment program (graduated) or left Against Medical Advice was recorded. The time of testing was also recorded as a number relative to the time of discharge in days.

### 2.4. Analysis

#### 2.4.1. Speech Recognition

Audio responses were recorded continuously throughout the Stroop task by the microphone built into the smart device, sampled at 16,000 Hz and saved in a .flac-format for further processing (Figure 1B). Voice response onsets were automatically timestamped at 10 milliseconds (ms) increments by an in-house developed automatic speech recognition model using the Kaldi speech recognition toolkit [26]. Stimulus on-screen onset was also timestamped, and the response latency was derived by calculating the duration between stimulus- and response timestamps. The language model was specifically tuned to recognize the relevant words in the Stroop task (i.e., the color words). This technique allowed us to take advantage of knowledge about the context of the spoken utterances, namely that words such as “**GREEN**” and “**RED**” were more likely to occur than “**CAR**” or “**SPOON**”, thus increasing the accuracy of the word recognition. Performance was evaluated by comparing machine transcripts to 175 manually transcribed recordings, and the word error rate for the recognizer was calculated at 6.26%. This was considered highly accurate and determined to be acceptable.

As a consequence of using automatic speech recognition for detecting responses, conclusions regarding the presence and accuracy of responses may be confounded by the processes of the recognizer. If there was no response detected, this may have been due to no utterance being made, but it may also have been that the utterance was indistinguishable from background noise (i.e., the utterance was too weak or unclear to be detected as a word). Equally, a response detected as “incorrect” by the automated system may, in fact, be due to an incorrect word uttered (e.g., “**RED**” or “**TIGER**” when correct is “**GREEN**”), but it may also be due to an automatic speech recognition error (e.g., the correct utterance “**GREEN**” is recognized as “**BLUE**”) due to the way it is pronounced, registering falsely as an error. In order to have an accurate response detected, the response must be (i) the correct color word and (ii) clearly stated. Accuracy was then defined as (Number of correct responses detected)/(Total number of presentations). It is acknowledged that this approach is extremely conservative such that responses from participants with slurred or otherwise impeded speech could be excluded from this particular analysis. Only responses recognized as “Correct” by the automatic system were included for response time (RT) processing. To limit the effect of artifact outliers, responses of less than 200 ms and outliers of longer than 3SD (per group; 1.61 s for patients and 1.36 s for nonpatients) were removed, as these were considered task-unrelated behavior.

#### 2.4.2. Performance Analysis

In order to extract a detailed description of response patterns on the Stroop task, we derived general metrics of performance alongside the conflict-related metrics that specifically assay attentional control. General performance metrics were processing speed, as measured by RT latencies (in milliseconds after stimulus onset), processing efficiency and central nervous system integrity, as measured by intra-individual variability of RTs [13], operationalized here as the SD of RT divided by the mean of RT and expressed as a percentage, i.e., the coefficient of variation of RTs, and accuracy (as a percentage of correct responses).

The specific goal of using the Stroop paradigm was to assess how conflicts between presented colors and word categories (i.e., congruent/animal-words/incongruent) affect the latency of responses. In order to deconstruct the different aspects of word processing, we calculated word category conflict effects in two related measures: (i) Interference, expressed as the difference between the mean response time of the incongruent trials and the mean response time on purportedly neutral trials, and (ii) Facilitation, expressed as the difference between the mean response time of the congruent trials and the mean response time of the color-neutral animal-words trials. Negative word category effect scores were set to zero to address nonlinearity effects in modeling.

Traditionally the focus of Stroop response analysis has been on when responses occurred (i.e., latencies), but digital recordings also allow for analysis of how a spoken response is uttered (i.e., acoustic properties). A variety of features are possible to extract from recordings, but we demonstrate this concept by measuring response word duration, namely the time span between the start of an utterance to the subsequent silence after it (Figure 1B).

#### 2.4.3. Statistical Methods

The statistical significance between groups and conditions (i.e., present or not) was assessed with chi-square, *t*-tests and analysis of variance, as well as multilevel modeling (MLM) performed with the ”lmer” package, all implemented in the R programming language. A broad exploration was conducted, and as such, marginally significant differences should only be considered suggestive. The distribution of the resulting RT data was, as expected, non-normal and ex-Gaussian, but we nonetheless considered parametric tests appropriate (and analyses of log-transformed, standardized response times were additionally performed but did not affect conclusions). Test-retest reliability across the five sessions was assessed with intraclass correlations (ICC (3, k)) using the R-package “psych” [27]. For MLMs, the participant was included as random effects in the model. Model fit was evaluated by comparing the full model to that of random intercepts using chi-square statistics. Independent variables were grand mean centered or dummy-coded (if binary). All data were trimmed such that values exceeding 3.5 SD from the grand mean were replaced with values of 3.5 SD from the grand mean.

## 3. Results

### 3.1. Data Considerations

**Figure 2:**
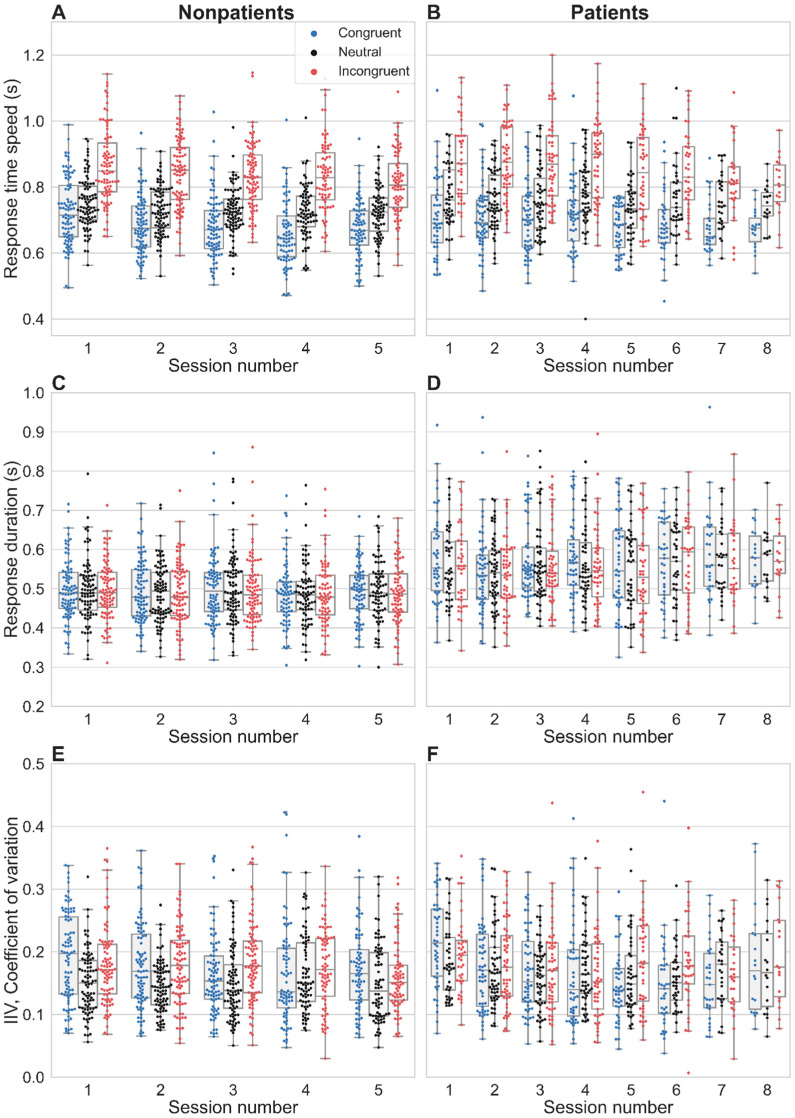
Estimates of average Stroop performance per session (k = 799) for incongruent (red), congruent (blue) and neutral (black) stimulus conditions. Panel (**A**): For nonpatients, response time speed was clearly slower for incongruent conditions versus neutral, the effect known as Interference. Speed was also faster for congruent conditions versus neutral, the effect known as Facilitation. Panel (**B**): The same pattern was found for patients, and patients were slower overall compared to nonpatients. Panel (**C**): Response duration, a parameter of word stress, did not differ between conditions for nonpatients. Panel (**D**): Utterances from patients had a longer duration than those from nonpatients but did not differ between conditions. Panel (**E**): Being able to keep consistent response times regardless of stimulus condition is a hallmark of good Stroop test performance. Intra-individual variability, represented by the coefficient of variation of response times within a session, showed decent test-retest reliability but did not differ consistently between conditions. Panel (**F**): Intraindividual variability was only marginally larger in patients but showed an interesting pattern of increased coefficients of variation in patients that left treatment Against Medical Advice as they approached their discharge date (see Figure 3C).

Mobile Stroop testing was acceptable to the participants, and on average nonpatient and patient groups completed five (range = 1 to 9) and six (range = 1 to 10) testing sessions, respectively (see Supplemental Materials for frequency histograms). Response accuracy was high at 95% ± 8% & 86% ± 17% for the nonpatient and patient groups, respectively, numbers that included both true errors from the participants but also errors from the automatic speech recognition procedure. Generally, the Stroop performance features were not significantly associated with age, sex or race for either the nonpatient or patient groups. A zero-order correlation matrix of all Stroop features is presented in Supplementary Figure S2. Response time latency scores for the different stimulus conditions were highly correlated with each other but were non-redundant (*r* values > 0.88), indicating that the word categories (color-congruent, color-incongruent or animals) indeed affected performance differently. Response time data are presented in Figure 2 and Table 1.

### 3.2. Stroop Interference and Facilitation on Response Time Latency

The mobile experimental operationalization of the Stroop interference task successfully replicated the classic effects, namely that the meaning of a word interfered with the naming of the ink color. Response times were significantly slower when the colors and meanings of stimuli were Incongruent versus when the stimuli were Neutral animal words, both for nonpatient (0.862 ± 0.131 & 0.749 ± 0.092, respectively, *t* = 25.79, *p* < 0.001) and patient (0.926 ± 0.174 & 0.814 ± 0.142, respectively, *t* = 22.49, *p* < 0.001) groups. This difference between Incongruent and Neutral response time latencies is what traditionally has been called the “Interference effect”. The average absolute size of the Interference effect across sessions was 0.112 s for both patients and nonpatient groups (see Table 1). Conversely, response times were significantly faster for the Congruent conditions versus Neutral conditions for nonpatient (0.702 ± 0.109 & 0.749 ± 0.092, respectively, *t* = 14.58, *p* < 0.001) and patient (0.735 ± 0.121 & 0.814 ± 0.142, respectively, *t* = 16.73, *p* < 0.001) groups. This difference between Congruent and Neutral response time latencies is what has traditionally been called the “Facilitation effect”. The average absolute size of the Facilitation effect was 47 ms for nonpatients and 79 ms for patients (see Table 1). Finding these effects between conditions when data are combined across participants was encouraging but not sufficient to know if the resolution of the testing system is good enough to find meaningful effects on the level of the individual. Examining participant-level results, approximately 92% and 88% of individuals from the nonpatient and patient groups showed an Interference effect, and approximately 75% and 82% of individuals from the nonpatient and patient groups showed a facilitation effect.

As expected, nonpatients versus patients were significantly faster on all stimulus conditions (*t*’s > 2.37, *p*’s < 0.02; Feature scores averaged across sessions). Note that given the many differences in characteristics of participants between the two groups (e.g., age, sex, race), such comparisons were not the main purpose of this experiment. Even so, finding such differences holds the promise that the procedure may be able to detect disease-specific differences in a more matched sample. For nonpatients, men and women were comparable in overall response time, but women showed larger interference effects (*t* = 2.38, *p* = 0.02, d = 0.56). Age was associated with smaller facilitation effects (*r* = 0.24, *p* = 0.03).

### 3.3. Temporal Properties

The Stroop performance measures showed fair to good stability across five and eight sessions for the nonpatient and patient groups (Table 1). For Response time scores, Intra-Class Correlation (ICC) values ranged from 0.83 to 0.95, indicating relatively high, but not perfect, temporal stability. The measures of interference and facilitation showed lower temporal stability. None of the ICC values appreciably changed when the ICC values were recomputed, excluding the first session, suggesting that there were no major practice effects from the first to second sessions.

### 3.4. Convergence with Clinical Symptoms Rating Scales

For nonpatients, none of the Stroop measures were significantly related to self-reported symptoms, as measured using the Brief Symptom Inventory [25]. These null findings were demonstrated with both correlations, with scores averaged across sessions within participants (*r*’s < 0.16, *p*’s > 0.10), and with multilevel modeling, with session data nested within participants. Null findings were found with patients as well, such that Stroop measures were not significantly related to Brief Psychiatric Rating Scale factor scores [23]. An exception was that longer response times for the neutral condition were observed for patients with higher agitation and positive symptoms (*r*’s > 0.32, *p*’s < 0.05).

### 3.5. Convergence with Self-Reported Concentration and Helplessness

For both patients and nonpatients, Stroop performance was significantly related to aspects of clinical state at the time of testing using multilevel modeling. For nonpatients, increased self-reported concentration (0–100 on a digital slider scale in the mobile application) was associated with significantly slower response times for congruent and neutral conditions (B (SE) = −0.0044 (0.0016) & −0.0032 (0.0015), respectively, *t*’s = 2.84 & 2.08, respectively, *p*’s < 0.05). Coefficients for the incongruent, facilitation and interference scores were not significant. For nonpatients, increased self-reported helplessness was not significantly associated with Stroop performance.

For patients, increased self-reported concentration was associated with slower response times for the congruent condition (B (SE) = −0.0036 (0.0016), *t* = 2.28, *p* < 0.05). They were also associated with decreased facilitation effects (B (SE) = −0.0027 (0.0012), *t* = 2.30, *p* < 0.05). Coefficients for the incongruent and for facilitation and interference scores were not significant. Increased helplessness was associated with slower response times for the congruent condition (B (SE) = −0.0036 (0.0016), *t* = 2.28, *p* < 0.05), but not the other conditions.

### 3.6. Relation to Treatment Outcome

**Figure 3:**
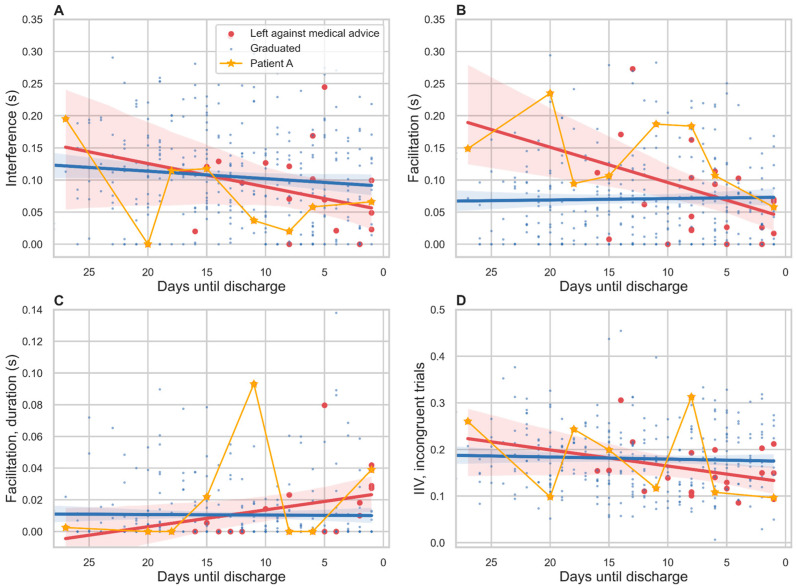
Scatterplots illustrating the exploratory analysis of relationships between Stroop scores and the time until discharge from the treatment facility. Most patients finished treatment as planned (Graduated; blue), while some left treatment Against Medical Advice (red). The orange line presents data from one individual patient to demonstrate how variable measurements can be over time within an individual. Panel (**A**): As patients approached their discharge date, interference effects tended to be smaller. Panel (**B**): Patients leaving Against Medical Advice showed decreased facilitation effects as they neared their discharge, while those who graduated from treatment did not. Panel (**C**): There was an interaction for facilitation response durations such that patients leaving Against Medical Advice showed greater facilitation effects as they neared their discharge. Panel (**D**): Response time variability on the incongruent trials increased as patients approached their discharge date, more so for patients who left Against Medical Advice. This is the most difficult condition containing the Stroop color-word conflict, and this finding holds some promise that a simple variability measure can show value in clinical settings.

Average response times (averaged across sessions) were not statistically different between patients that graduated from treatment versus those that left Against Medical Advice (*t*’s < 1.64, *p*’s > 0.10). Multilevel modeling revealed that as patients approached their discharge date, their response times for the incongruent (B (SD) = −0.16 (0.03), *t* = 5.21, *p* < 0.05) and neutral (B (SD) = −0.07 (0.03), *t* = 2.29, *p* < 0.05) conditions got longer. Moreover, their interference (B (SD) = −0.15 (0.05), *t* = 3.25, *p* < 0.05) effects tended to be smaller (Figure 3A). A significant interaction was seen for facilitation such that patients leaving Against Medical Advice showed decreased facilitation effects as they neared their discharge (B (SD) = 0.11 (0.05), *t* = 2.05, *p* < 0.05; Figure 3B).

### 3.7. Response Duration

Measures of response word duration were relatively stable over time, with ICC scores ranging from 0.76 to 0.87 across both groups (Table 1). Patients versus nonpatients showed longer response word durations for each of the congruent (0.561 s vs. 0.493 s), incongruent (0.551 s vs. 0.492 s) and neutral (0.555 s vs. 0.493 s) conditions (63 ms difference across conditions, *t*’s > 6.02, *p*’s <0.001). Facilitation and interference effects were not robustly observed in response durations, with differences between average durations for conditions being just 5 ms between congruent vs. neutral and 2 ms between neutral vs. incongruent. This difference is well below the 10 ms resolution/quantization error of the response latency measurements. Even so, given a large number of samples, it was possible to find a statistically significant difference where patients showed significantly shorter responses in the congruent (0.561 s) versus neutral (0.555 s) conditions (6 ms difference, *t* = 22.49, *p* < 0.05). Nonpatients showed no differences in response durations between congruent/incongruent and neutral conditions (*t*’s < 1.08, *p*’s > 0.30). Facilitation and Interference scores, computed as a function of response duration, were not stable over time (Table 1).

Response duration scores were not related to clinical symptom rating scales for either group and, with several exceptions, were unrelated to self-reported concentration and helplessness. Shorter response durations for the congruent condition were related to higher concentration for nonpatients (B (SD) = 0.0035 (0.0016), *t* = 2.16, *p* < 0.05) and lower helplessness for nonpatients (B (SD) = 0.0037 (0.0017), *t* = 2.13, *p* < 0.05). For patients, greater facilitation effects of response duration were related to decreased concentration (B (SD) = −0.0027 (0.0012), *t* = 2.30, *p* < 0.05) and increased helplessness (B (SD) = 0.0037 (0.0014), *t* = 2.70, *p* < 0.05). With respect to treatment outcome in patients, a significant interaction was seen for facilitation response durations such that patients leaving Against Medical Advice showed greater facilitation effects as they neared their discharge (B (SD) = −0.13 (0.05), *t* = 2.66, *p* < 0.05, Figure 3C). No other significant relationships between response duration scores and treatment were observed.

### 3.8. Intra-Individual Variability

Measures of intra-individual variability were only modestly stable over time, with ICC scores ranging from 0.39 to 0.80 across both groups (Table 1). Patients versus nonpatients showed greater variability for the incongruent and neutral conditions (*t*’s > 2.03, *p*’s < 0.02) but not the congruent condition (*t* = 0.57, *p* = 0.19).

Intra-individual variability scores were generally not related to clinical symptoms for either group, though for nonpatients, higher scores were related to more severe psychotic, somatic and anxiety symptoms for the congruent, incongruent and incongruent conditions, respectively (*r*’s > 0.18, *p*’s < 0.05). Variabilities were also generally not related to the clinical state of nonpatients. For patients, increased helplessness was associated with greater variability for the incongruent condition (B (SD) = 0.0037 (0.0018), *t* = 4.15, *p* < 0.05). With respect to treatment in patients, those that went Against Medical Advice had significantly greater intra-individual variability scores from the congruent condition overall (0.17 (0.07), *t* = 2.25, *p* < 0.05). As patients approached their discharge date, their variability in the incongruent (−0.15 (0.05), *t* = 3.06) and neutral (−0.13 (0.05), *t* = 2.95, *p* < 0.05) conditions tended to increase. A significant interaction was seen for the incongruent condition such that patients leaving Against Medical Advice showed greater variability on the incongruent condition as they neared their discharge (B (SD) = −0.13 (0.05), *t* = 2.66, *p* < 0.05, Figure 3D).

## 4. Discussion

When administering the classic Stroop interference task as a brief (90 s long) version via smart devices, it was possible to use speech recognition software for timestamping responses and measuring robust effects of word categories on color naming, with the expected pattern of delayed responses in the color-conflict condition. Healthy participants had faster, less variable, and more accurate responses as compared to patients. There was no difference between groups on the traditional Stroop Interference measure, but patients did show larger Facilitation effects, a pattern also found in other clinical conditions [18,19,20,22]. Speech processing tools additionally revealed differences in word utterance duration, where response word utterances were generally longer in patients. An examination across multiple days revealed that even though the word category effects were robust—in the sense that they were not extinguished by practice—the traditional Stroop Interference and Facilitation scores showed lower test-retest reliability and were not consistently present when looking at individual participants. Excitingly, these sizes of the measured effects are similar in magnitude to previous findings of clinical relevance (i.e., 50–400 ms interference by words of affective salience [5]), providing a proof-of-concept for future mobile remote administration that can leverage voice to assay strong effects on attentional control and bias. The well-established Stroop paradigm, therefore, appears to be suited as a flexible and scalable platform for future investigations using smart devices and fast internet-based analysis and feedback.

### 4.1. Constraints on the Temporal Resolution of Automated Speech Analysis

**Figure 4:**
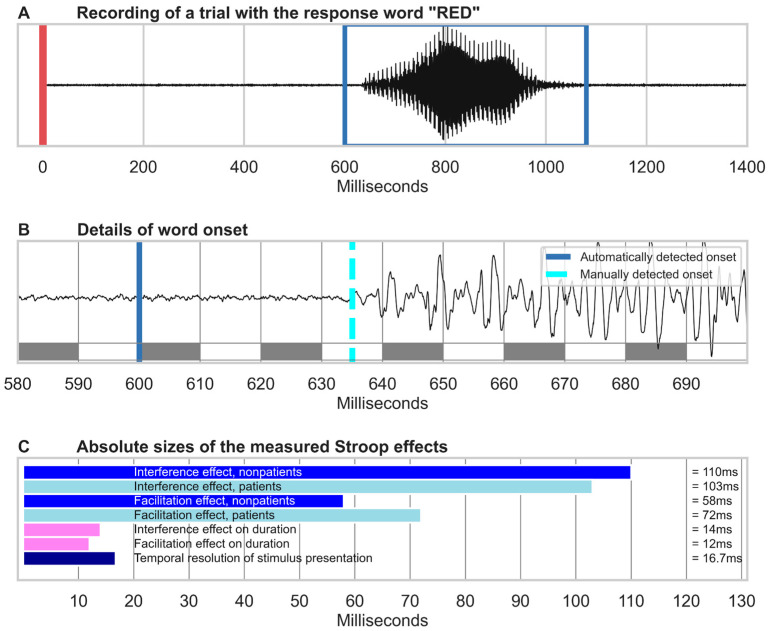
Illustration of the relationship between the temporal resolution of automatic speech recognition, the soundwaves of speech and the sizes of the Stroop effects and group differences. Panel (**A**): The audiogram of a trial where a male participant responded with the word “red”. Stimulus onset is marked with a red vertical line. Automatically detected response onset and offset marked in blue. Panel (**B**): Looking closer at the sound pressure oscillations at the onset of the word in detail, it was evident that the automatically detected onset (blue) was about 30 ms earlier than the manually detected onset (cyan). For the whole session (32 trials), the average difference between manual and automatic timestamps was 36 ms (SD = 12 ms), indicating a bias towards consistently early automatic timestamping. Such a bias should be formally examined in future studies. In seeking millisecond precision, there is a need for a robust procedure to determine where on the waveform the “onset” is to be stamped. Very few peaks of the waveform fall within each frame or “chunk” of the 10 ms temporal resolution, illustrated by alternating white and gray sections. Panel (**C**): The average sizes of the Stroop Interference and Facilitation effects for nonpatients (dark blue) and patients (light blue) are illustrated as horizontal bars on the same scale as the temporal resolution illustration in panel (**B**). The sizes of these traditional effects are several times larger than the resolution. The difference in the Interference effect between groups was lower than the resolution of the system, on the order of one or only a few vibrations of the vocal cords of this male speaker. Differences in Facilitation were three to four times larger than the resolution; it was also on this feature that it was possible to find statistically significant differences between groups. Interference and facilitation effects on duration were close to the 10 ms resolution (light purple). The last bar (dark purple) illustrates the relative size of the crucial ±16.7 ms resolution of stimulus presentation, a consequence of the 60 Hz refresh rate that is common for screens of mobile devices. Modeling differences in response times at the limit of the resolution of the measurement system is challenging. Increasing the number of trials will not change this fundamental issue of temporal resolution, but new methods for presenting stimuli and timestamping responses might.

Differences in response times must be of a certain magnitude to be reliably detected with automatic speech recognition. Parsing the minuscule differences between stimulus conditions and participant groups were challenged by both technical and biological constraints. Even though the use of voice recordings allowed bypassing several possible sources of measurement error (e.g., 4–30 ms variability in commonly employed USB keyboards, 60 Hz/16.7 sample rate on touchscreens), there were also limits to the resolution with which one could meaningfully timestamp the onset of a vocal response. For example, at the time frame of 10 ms (i.e., typically the highest resolution of automatic speech recognition timestamping; see Figure 4), low-pitch speech information with a frequency of around 125 Hz (male voice) would only provide 1–2 main fluctuations in air pressure level from the corresponding vibrations of the vocal cords. Also, considering the variability in the muscular responses in the thorax and larynx needed to produce and release airflow, it is evident that when we are examining differential phenomena that are on the order of 30–40 ms (such as the differences in facilitation effects in the current paradigm), we may be close to the limit of what effects can be parsed with verbal behavior. Even so, we argue that the current approach of collecting high-quality audio responses with mobile devices holds promise. Methods for timestamping responses can be improved, and unlike the specialized hardware solutions for computerized cognitive experiments in laboratories, it is now possible to create experiments where the hardware apparatus can be practically equal across millions of participants and in principle can nurture equity in terms of the availability of assessment tools. Naturally, there remain several issues to be overcome in the long term, like ensuring equal access to devices, but focusing on a subset of highly popular device types and optimizing temporal precision for these systems can provide unprecedented uniformity of measurement errors across data collection situations.

### 4.2. Future Improvements

Several issues with the design of the current experiment may be improved in future implementations in order to increase the generalization of findings beyond the demonstration of the effectiveness of speech processing technologies. First, the resolution of the timestamping of response onsets needs to be increased, and the timing of the on-screen presentation of stimuli must be validated. Modern software packages and online solutions for psychological and psychophysical experiment generation on desktop/laptop computers have recently been extensively examined and found to provide decent timing performance themselves (precision < 10 ms) when ignoring problems with attached hardware like displays and keyboards [28]. Errors in the estimate of response times can partly be counteracted by an increased number of trials per participant in an experimental paradigm such as the Stroop task, where some sources of variability will cancel out when response time estimates are subtracted to get difference-scores. However, during the development of the assessment tool, it quickly became evident that there was an optimal length of a session and individual tasks that ensured people would even use the remote self-administered system. Put differently, adding more trials would not be feasible from a usability perspective and ultimately would lead to fewer data. For an extended discussion of the usability aspects of mobile Stroop testing, see [9]. As such, there are limitations to how much resolution can be increased by increasing trials (i.e., reducing the quantization error by oversampling and averaging). This being said, the issue of the automatic speech recognition temporal resolution may be considered less problematic than the large variability (up to 11–73 ms [29], 20–40 ms [30]) in response time that can stem from the rather common practice of using various off-the-shelf keyboards for recording responses. Second, the short time span of testing sessions (i.e., five to eight days) was insufficient to provide intra-individual comparison between periods with disordered states (e.g., psychosis, mania) versus stable states. With more time points, the longitudinal nature of our data could be more suited for robust examination using latent change scores and latent growth curve models [31]. Even so, combining data from a participant over five to eight days should be highly suitable to measure differences on a week-to-week basis. For example, it would allow for robust measurements of potential differences in performance before and after initiating a pharmacological intervention or comparison between clinically stable phases for a patient in an outpatient setting versus when the same patient is hospitalized due to relapse. It is unknown whether such within-person effects will be large enough to overcome the aforementioned lower bound of effect sizes possible to parse using voice responses. Third, the type of stimulus set used, where both attentional control and attentional bias due to the salience of words (i.e., animal categories) affect performance, presents a complicated situation with many degrees of freedom for interpretation. It does, however, reveal the potential for suitable paradigms to parse both cognitive abilities and personal levels of word salience. On a more technical note, the actual colors used might affect timestamping accuracy, where utterances of a color like “purple” that starts with a splosive (a much more abrupt onset of the airflow and resulting sound compared to the “red” in Figure 4) may be more accurately timed. The design in the current proof-of-concept study employed a generic and well-explored set of stimuli, but the technology allows for vastly more complex, tailored, and adaptive approaches. Ultimately, the current ‘one-paradigm-fits-all’ approach may not be sufficiently effective, and future methods could employ personalized adaptive paradigms able to tailor stimulus materials to more effectively gauge individual levels of performance and longitudinal change. Finding the right stimulus material can also be critical to reaching effect sizes that are detectable with current limitations to response timing resolution. Such adaptive paradigms may also be configured to be more entertaining to the user, thus allowing more trials and more robust metrics.

The demonstration of differences in word utterance durations holds the promise of a multitude of new ways one can extract information from responses in spoken assessment tasks. Duration has been found to be an acoustic correlate of word stress, or emphasis, across a large number of languages, over and above other traditional acoustic parameters such as fundamental frequency, intensity, formants and spectral tilt [12]. Response properties are not limited anymore to simple accuracy and timestamping measurements, as it is now technologically feasible to assay the expression of affective states using prosodic elements of speech such as sound pressure and pitch. Indeed, we had previously found that using a machine learning approach on over 6000 acoustic parameters derived from this seemingly innocuous Stroop task were remarkably more direct assays of affective states as compared to when such measures were derived from story retelling, picture description and even verbal self-reports on the subjective state (i.e., “How do you feel today?”) [32]. Put differently, affect measures derived from a person’s utterance of a color word can provide crucial and clinically relevant signals in an inherently non-threatening manner, in that confrontation of potentially arousing or debilitating topics can be avoided. Naturally, acoustic metrics of affective states can provide a more complete picture of the neurocognitive state of the individual, as emotional valence and levels of arousal can have a modulating effect on cognitive performance [33]. Additionally, this can be expanded by combining the method with other objective measures, as the Stroop test is ideally suited for using pupillometry as a biomarker for arousal [34] and task demands or mental effort [35]. By mapping the individual distribution of performance and relevant biomarkers over time, these technologies can enable us to assess the dynamic effects of emotional states on cognitive functions.

## 5. Conclusions

Mobile technology and new automated analysis methods offer plenty of opportunities but warrant a careful examination of what they can deliver. In the case of the Stroop task, it is possible to sample repeatedly and remotely, but there is a limit to how many trials participants are willing to do in a session before boredom sets in and lowers usability. This limit has effects on the resolution of the measurement system. When simply increasing trials is not an option, it will be crucial for the field to carefully investigate how the precision and resolution of time can be increased for conducting research like this on the devices that are de-facto available, namely common smartphones. Timing properties have been examined extensively for desktop computers and web-based solutions [28,36], and such efforts, including the use of oscilloscopes with light and sound sensors, should be extended to mobile solutions. As for the voiced-based Stroop task, there are even biological limitations. The brain activity that we are ultimately interested in studying needs to affect complex motor output to set vocal cords vibrating. These vibrations will give limits to the resolution with which we are able to describe our signal, the air-pressure waves of human speech.

In conclusion, we have shown that an adaptation of a brief spoken Stroop paradigm implemented on a smart device can provide an experimental framework to enable the identification of specific attentional biases and assessment of the ability to control behavior. These functions are at the core of most cognitive processes critical for our everyday life. The methodology utilized in this study, both in terms of stimulus presentation and vocal response processing, opens up new venues for longitudinal behavioral assessments in humans, as long as the meaningful clinical effects are large enough. Indeed, technology is changing the nature of behavioral assessment and research [37], and the resulting models of brain function and dysfunction bring the promise of personalized medicine closer to realization.

## Figures and Tables

**Table 1:** Response time latencies, Stroop effect scores, response durations and coefficients of variations.

	Nonpatients (N = 113)		Patients (N = 85)		ICC
	M	SD	M	SD	
Speed (s *)					
Overall	0.745	0.079	0.766	0.092	0.83
Congruent	0.684	0.097	0.697	0.098	0.80
Neutral	0.731	0.077	0.762	0.095	0.82
Incongruent	0.839	0.112	0.860	0.122	0.85
Stroop Effect scores (s *)					
Interference	0.110	0.078	0.103	0.076	0.66
Facilitation	0.058	0.053	0.072	0.066	0.78
Duration of utterance (s *)					
Overall	0.495	0.078	0.563	0.100	0.57
Congruent	0.496	0.082	0.568	0.106	0.56
Neutral	0.495	0.081	0.558	0.099	0.39
Incongruent	0.494	0.084	0.555	0.101	0.53
*Coefficient of variation*					
Overall	0.190	0.045	0.198	0.052	0.68
Congruent	0.172	0.069	0.171	0.076	0.76
Neutral	0.151	0.053	0.169	0.059	0.70
Incongruent	0.174	0.062	0.179	0.071	0.43

* Response timestamps were derived with a 10 ms resolution. Therefore, there were not sufficient significant digits to report the customary millisecond values (see Discussion). The sub-resolution digit (i.e., third) is presented in a smaller font size to emphasize the problem with interpreting millisecond differences. ICC refers to Intra-class Correlation Coefficients.

## Data Availability

A de-identified subsample of the data, along with code illustrating the core analysis procedure, will be available on reasonable request from the corresponding author (Terje B. Holmlund).

## Supplementary Materials

The following supporting information can be downloaded at: https://www.mdpi.com/article/10.3390/brainsci13030442/s1, Figure S1: Panel (A). Histogram of sessions completed for nonpatients; Panel (B). Histogram of sessions completed for patients; Figure S2: Panel (A). Zero-order correlation plot of Stroop features for patients; Panel (B). Zero-order correlation plot of Stroop features for patients.
