# Supplementary material for: Using Automated Speech Processing for Repeated Measurements in a Clinical Setting of the Behavioral Variability in the Stroop Task
Source: Brain Sci. 2023 Mar 4;13(3):442. doi: 10.3390/brainsci13030442 (PMC10046258; doi:10.3390/brainsci13030442)

Supplemental Materials

Figure S1 Panel A. Histogram of sessions completed for nonpatients.

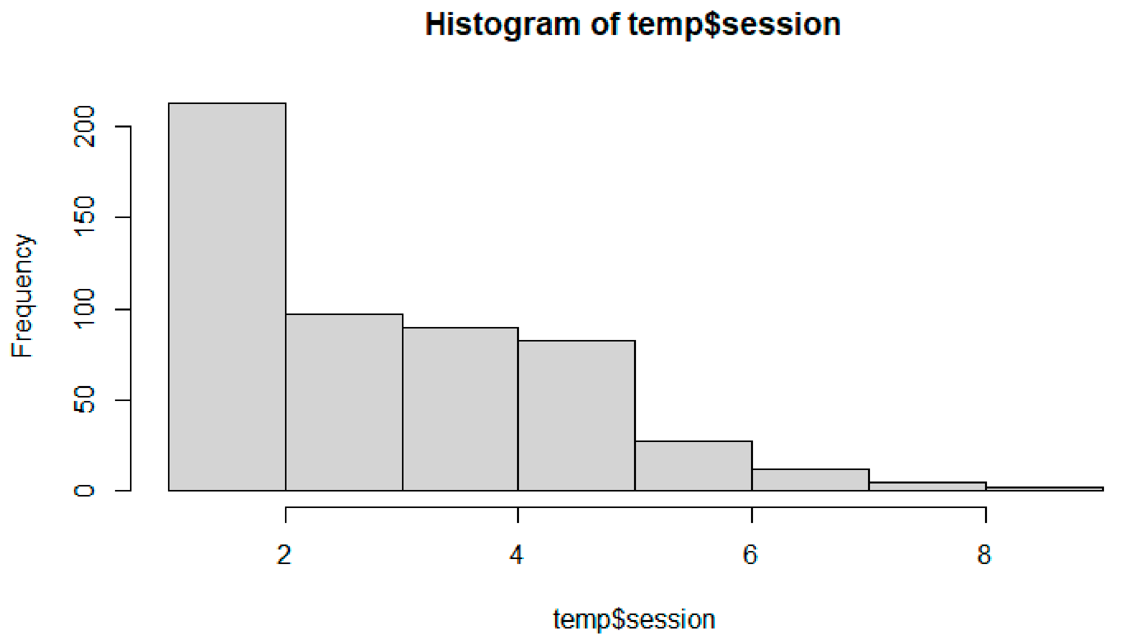

Figure S1 Panel B. Histogram of sessions completed for patients.

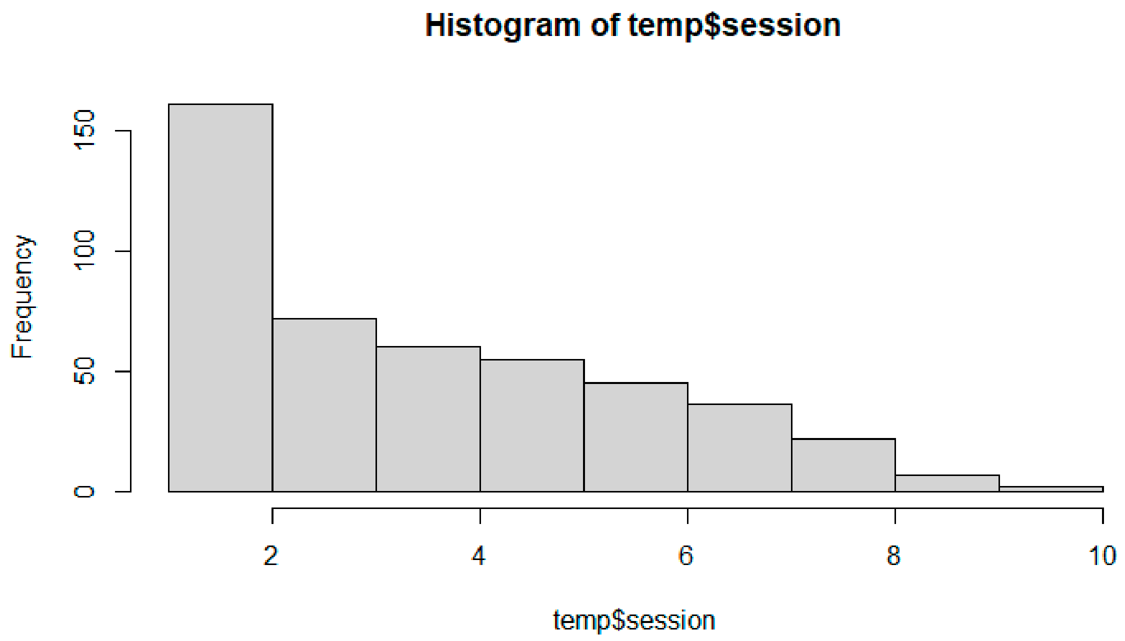

Figure S2 Panel A. Zero-order correlation plot of Stroop features for nonpatients

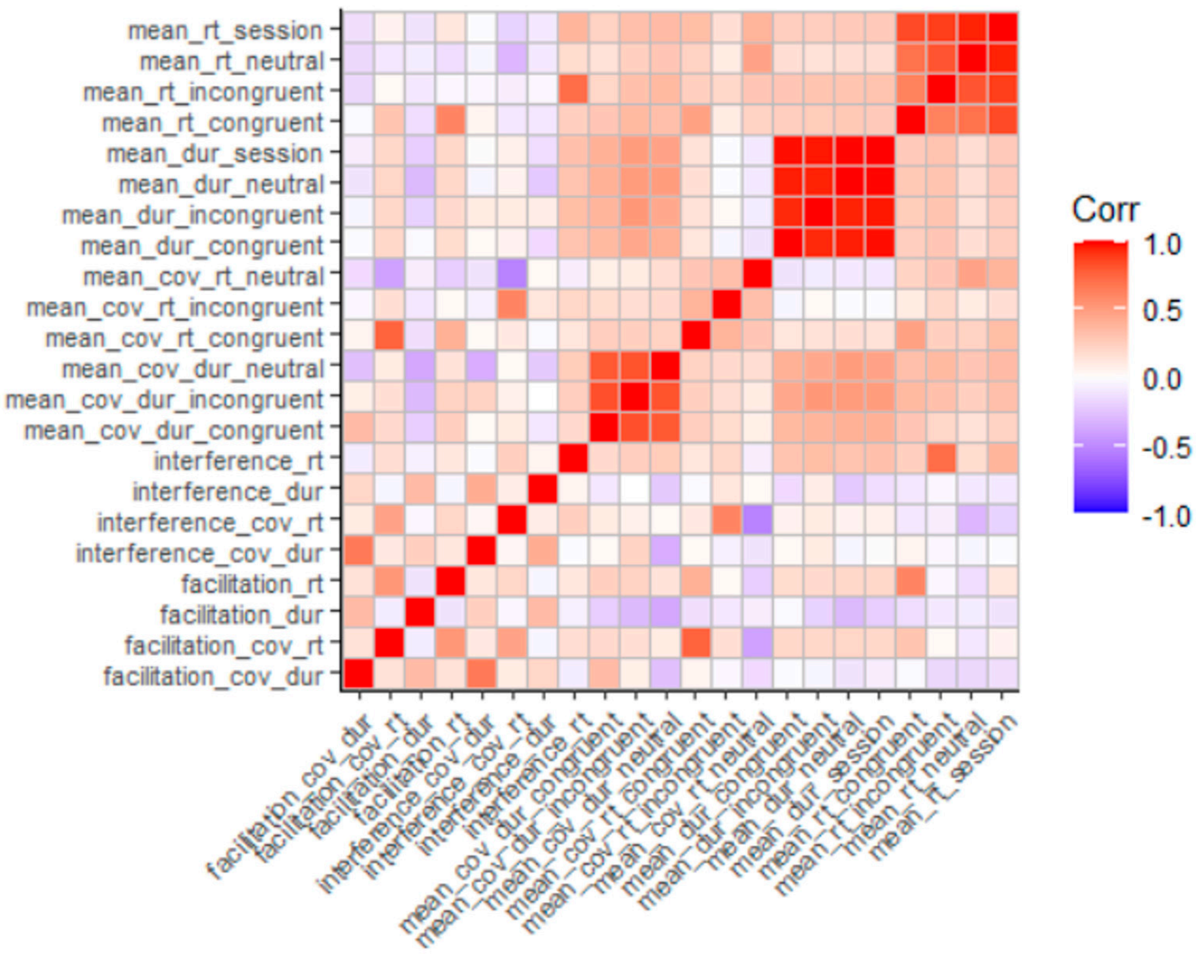

Figure S2 Panel B. Zero-order correlation plot of Stroop features for patients

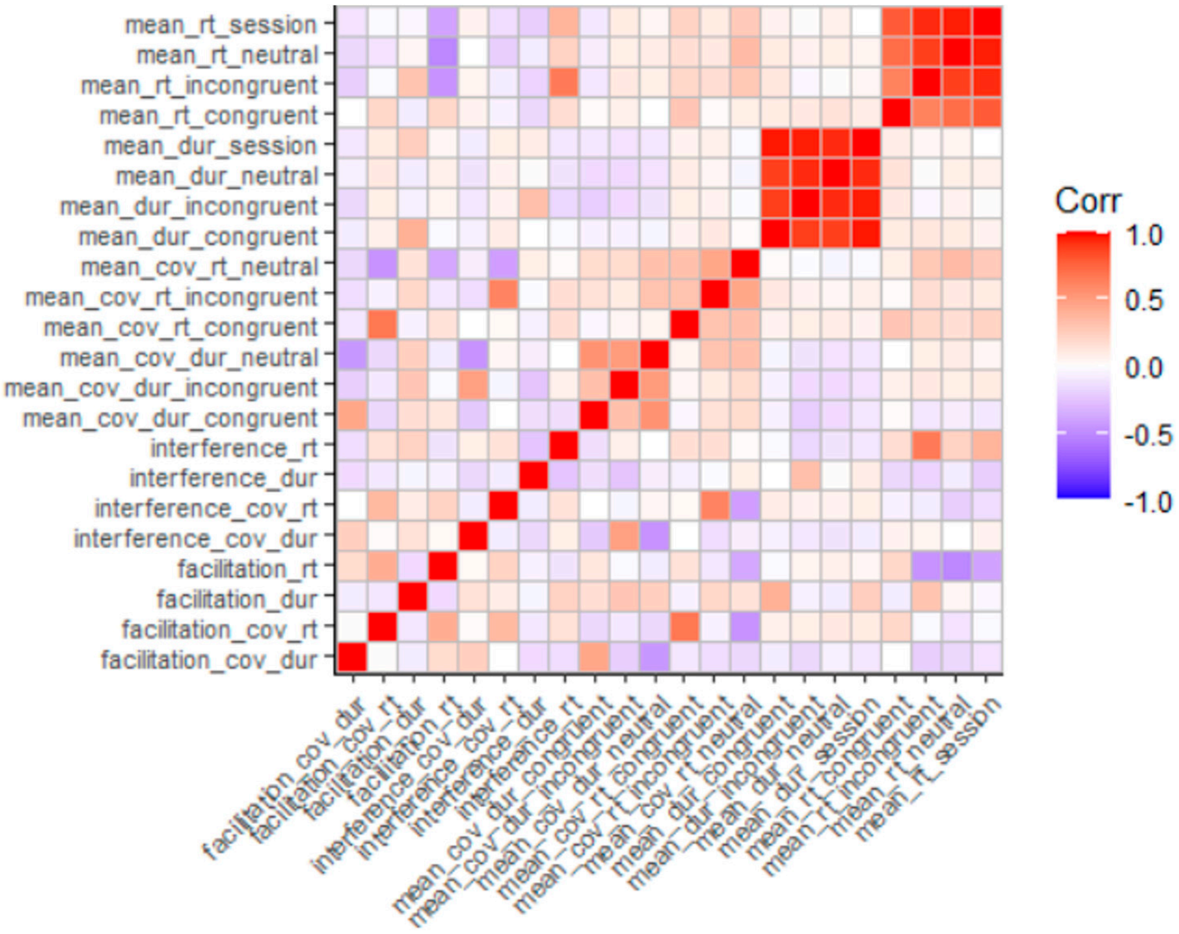

Supplement: Supplementary file 1 [file brainsci-13-00442-s001.zip › brainsci-2223379-supplementary.pdf]
